# Clinical and morphological predictors of metastasis in papillary thyroid microcarcinoma

**DOI:** 10.3389/fendo.2025.1637850

**Published:** 2025-10-10

**Authors:** Andrey Varlamov, Alina Elfimova, Alina Matrosova, Nano Pachuashvili, Ariya Dzodzaeva, Konstantin Slashchuk, Petr Nikiforovich, Dmitry Beltsevich, Vladimir Vanushko, Liliya Urusova

**Affiliations:** ^1^ Department of Fundamental Pathology, Endocrinology Research Centre, Moscow, Russia; ^2^ Institute of Clinical Morphology and Digital Pathology, I.M. Sechenov First Moscow State Medical University (Sechenov University), Moscow, Russia

**Keywords:** papillary thyroid microcarcinoma, lymph node metastasis, histopathology, risk factors, psammoma bodies, tumor budding

## Abstract

**Introduction:**

Papillary thyroid microcarcinomas (PTMCs), defined as papillary thyroid carcinomas measuring ≤1 cm, are frequently diagnosed incidentally and are generally associated with favorable outcomes. However, a significant subset of patients develops regional lymph node metastases. This study aimed to identify clinical and morphological features associated with metastatic spread in PTMCs.

**Methods:**

A total of 100 cases were retrospectively analyzed, including 50 with confirmed lymph node metastases and 50 without. A detailed histological assessment included evaluation of tumor subtype, nuclear features, presence of psammoma bodies, tumor “buds,” fibrosis, and other parameters. Clinical variables such as age, sex, body mass index, and presence of the BRAF V600E mutation were also considered.

**Results:**

Logistic regression revealed that younger age, classical histological subtype, tumor “budding,” and presence of psammoma bodies were independently associated with an increased risk of regional metastases, while concomitant multinodular hyperplasia was negatively associated.

**Discussion:**

The combination of these features may enhance risk stratification and guide the clinical management of PTMC patients, including decisions on surgical extent and follow-up strategies. Our findings support the relevance of comprehensive histological and clinical evaluation in predicting the metastatic potential of PTMCs.

## 1 Introduction

Thyroid cancer has been and remains the most common malignant neoplasm of the endocrine system (1, 2). Approximately 80% of malignant thyroid tumors are differentiated forms of cancer, the most frequent of which is papillary carcinoma (1). This histological type accounts for 50% to 80% of all malignant thyroid tumors, according to various data (3, 4). In many countries, recent years have seen an increase in the incidence of thyroid cancer, primarily due to the increased diagnosis of papillary carcinoma (5). However, this rise in thyroid carcinoma incidence has not been accompanied by an increase in tumor-specific mortality. This fact is mainly due to the more frequent diagnosis of papillary microcarcinomas, incidentally detected either during pathological examination of thyroid tissue removed for non-tumor pathology or during ultrasound examinations performed for reasons unrelated to thyroid pathology (6). Such tumors account for about 28% of diagnosed papillary thyroid carcinomas (4). Papillary thyroid microcarcinomas are defined as papillary carcinomas measuring no more than 1 cm, regardless of growth patterns (3, 7). Previously, terms such as “occult papillary carcinoma,” “latent papillary carcinoma,” “small papillary carcinoma,” “nonencapsulated thyroid tumor,” and “occult sclerosing carcinoma” were used for these tumors (8, 9). According to various sources, papillary thyroid microcarcinomas, which are clinically silent, may be found in 4–35.6% of cases during total histological examination of thyroid glands (4, 10, 11). The frequency of their incidental discovery in thyroid glands operated on for other diseases is directly proportional to the thoroughness of the histological examination (12). Researchers agree that the prognosis for patients operated on for papillary thyroid microcarcinomas is very good, with high recurrence-free and tumor-specific survival rates (12–14). Nevertheless, data from several studies indicate that the frequency of regional lymph node metastases in papillary thyroid microcarcinomas ranges from 17.1% to 49% (4, 14–16). It is known that a higher risk of metastasis is observed in tall cell, diffuse sclerosing, solid/trabecular, columnar cell, and hobnail subtypes of papillary carcinoma (17–21). Additionally, regional metastases are more frequently detected in pediatric patients (22). A higher risk of lymph node metastasis is also characteristic of patients with tumor invasion into lymphatic vessel lumens (23) and those with the *BRAF V600E* mutation in tumor tissue (24, 25). However, in practical work, situations often arise where metastatic involvement of regional lymph nodes is detected in patients who do not belong to known risk groups. Thus, identifying predictors of regional metastasis will allow for future optimization of clinical management protocols for such patients, refinement of the diagnostic criteria for papillary thyroid microcarcinoma, and consideration of reclassifying a portion of these tumors from malignant to neoplasms with limited malignant potential. This, in turn, would justify the implementation of active surveillance strategies and the incorporation of such an approach into clinical algorithms and guidelines.

The aim of the present study was to identify clinical and morphological predictors of regional metastasis in papillary thyroid microcarcinomas.

## 2 Materials and methods

### 2.1 Patients and samples

The study was conducted on surgical material from 100 patients aged 24 to 72 years (Me = 46 [34;61] years) operated on at the Endocrinology Research Centre (Moscow, Russia), for thyroid diseases between January 1, 2022, and September 1, 2023, with morphologically verified papillary thyroid carcinomas measuring ≤1 cm in greatest dimension (median = 0.7 cm; Q1 = 0.5 cm; Q3 = 0.9 cm). Exclusion criteria included age younger than 19 years and multifocal tumor growth. The study group comprised 50 patients with histologically verified synchronous metastases in regional lymph nodes (11 men, 39 women, aged 24 to 65 years). Among them, 45 patients had metastases only in the central compartment lymph nodes (pN1a according to TNM 8), and 5 patients had metastases in both the central and lateral compartments (pN1b according to TNM 8). The control group consisted of 50 patients without clinical or morphological evidence of metastatic involvement of regional lymph nodes, aged 25 to 72 years, including 8 men and 42 women. For the control group, there was also no evidence of regional metastasis development within 183 days after surgery.

Prophylactic lymph node dissection was performed only in patients presenting with clinical signs of lymph node involvement. In cases where the tumor was localized in one lobe of the thyroid gland and regional lymph node metastases were suspected in the central compartment (level VI), ipsilateral central lymph node dissection was carried out. Involvement of lateral neck lymph nodes was observed in four patients, who subsequently underwent lymphadenectomy of levels II to V; one patient underwent dissection of level III lymph nodes only.

In our study, none of the four patients with tumors located in the thyroid isthmus showed clinical evidence of regional lymph node involvement; therefore, intentional lymph node dissection was not performed. Nevertheless, in three of these patients, between two and four level VI lymph nodes, which were intimately adjacent to the isthmus, were unintentionally removed. In two of them, metastatic involvement of one lymph node was identified.

### 2.2 Morphological examination

The surgical material was fixed in 10% neutral buffered formalin (pH 7.4) for 18–20 hours, followed by sectioning, processing in a vacuum histoprocessor using a standard protocol, and embedding in paraffin blocks. For histological preparations, sections 3–4 µm thick were made. Then the slides were stained with hematoxylin and eosin according to the standard procedure.

**Figure 1:**
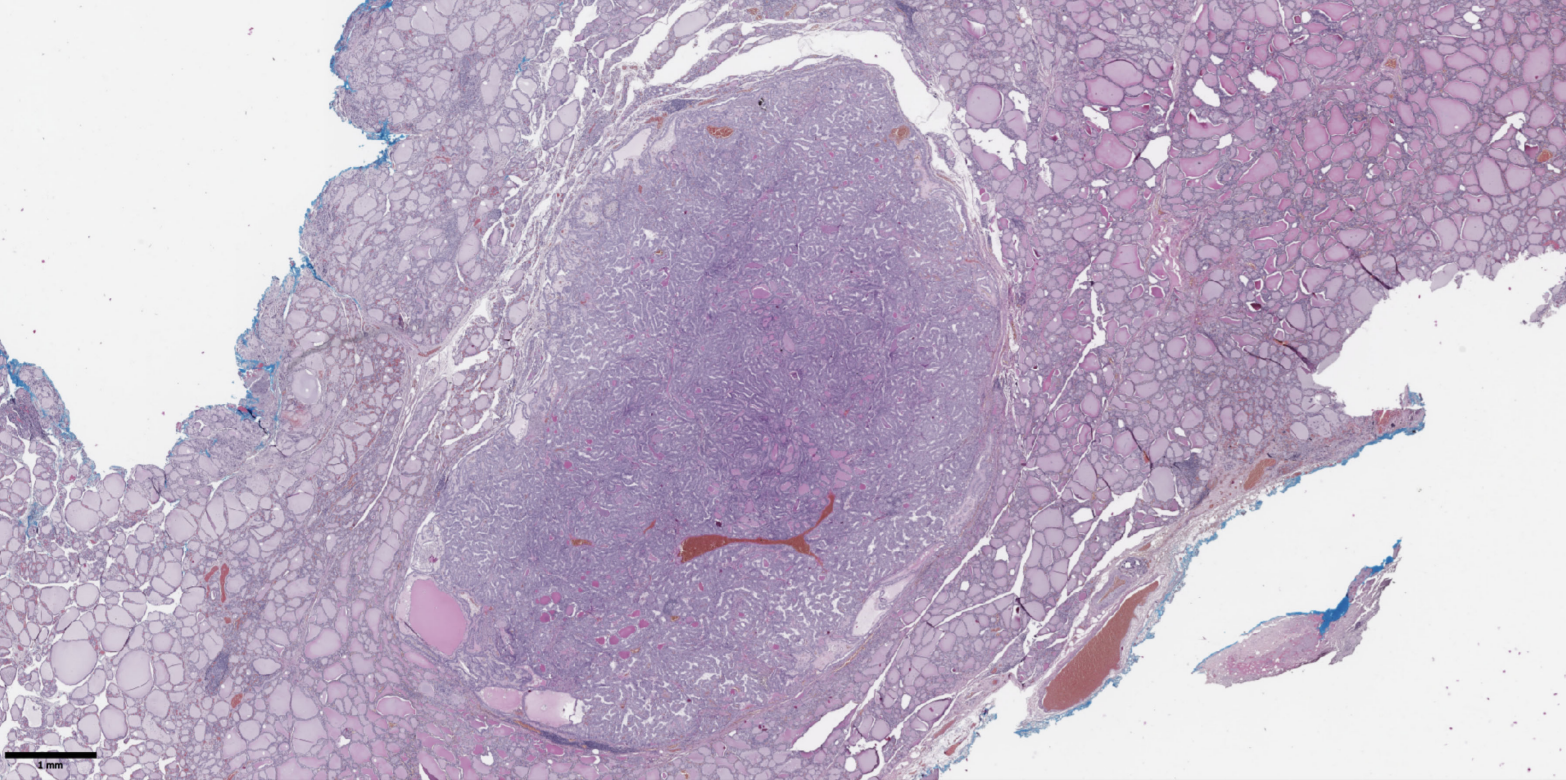
Histological image of papillary thyroid microcarcinoma. The tumor node is represented by papillary and follicular structures formed by cells with papillary nuclear features. Hematoxylin–eosin, х20.

**Figure 2:**
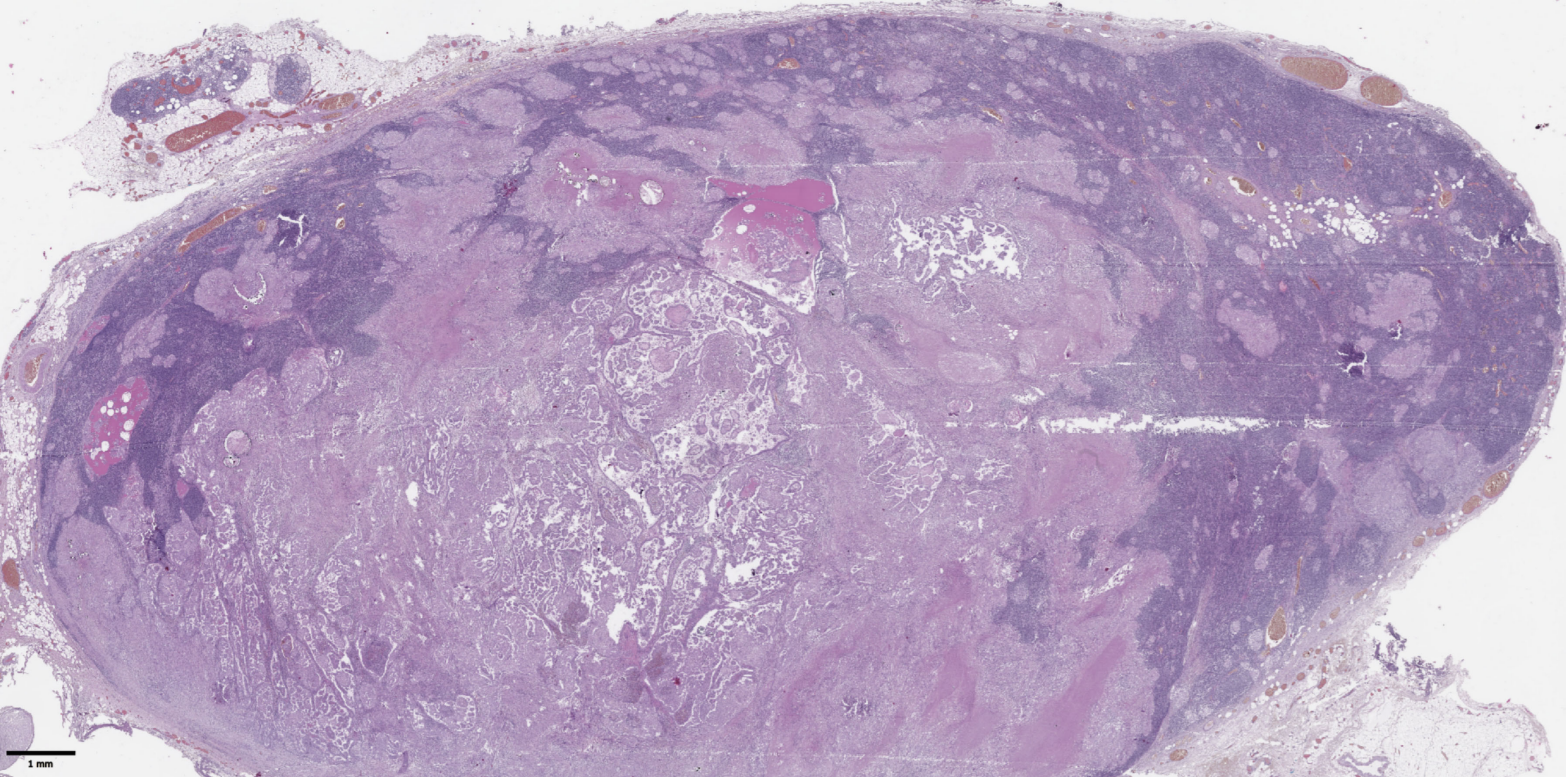
Histological images of metastases in regional lymph nodes of papillary thyroid microcarcinoma. Most of the lymph node tissue is replaced by a tumor. Hematoxylin–eosin, х30.

**Figure 3:**
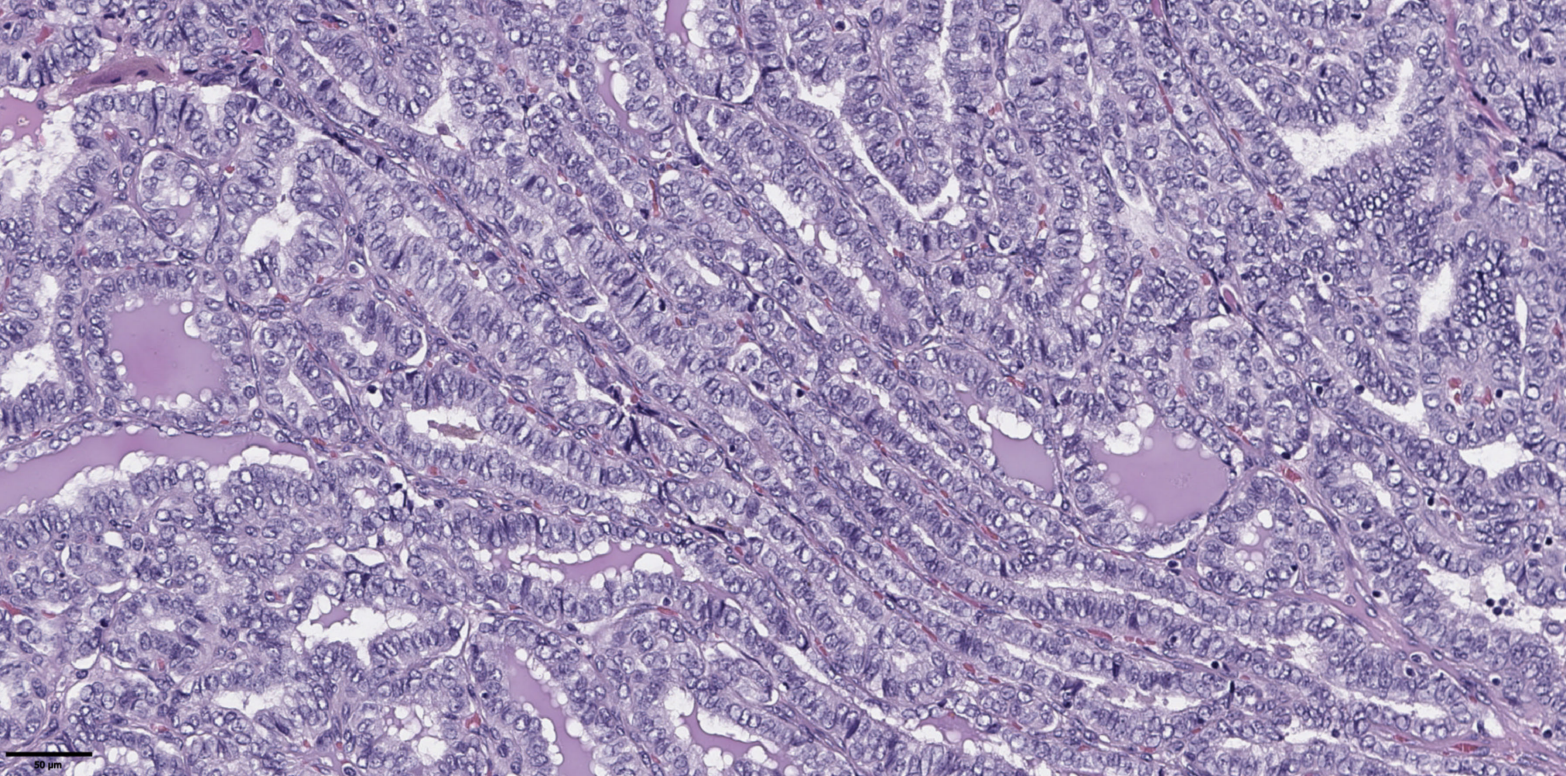
Histological images of papillary thyroid microcarcinoma, tall cell subtype. The tumor is composed of complex papillary and elongated trabecular formations that have a «tram-track» appearance. They are lined by crowded cells that have a height-to-width ratio of ≥ 3:1. Hematoxylin–eosin, х100.

**Figure 4:**
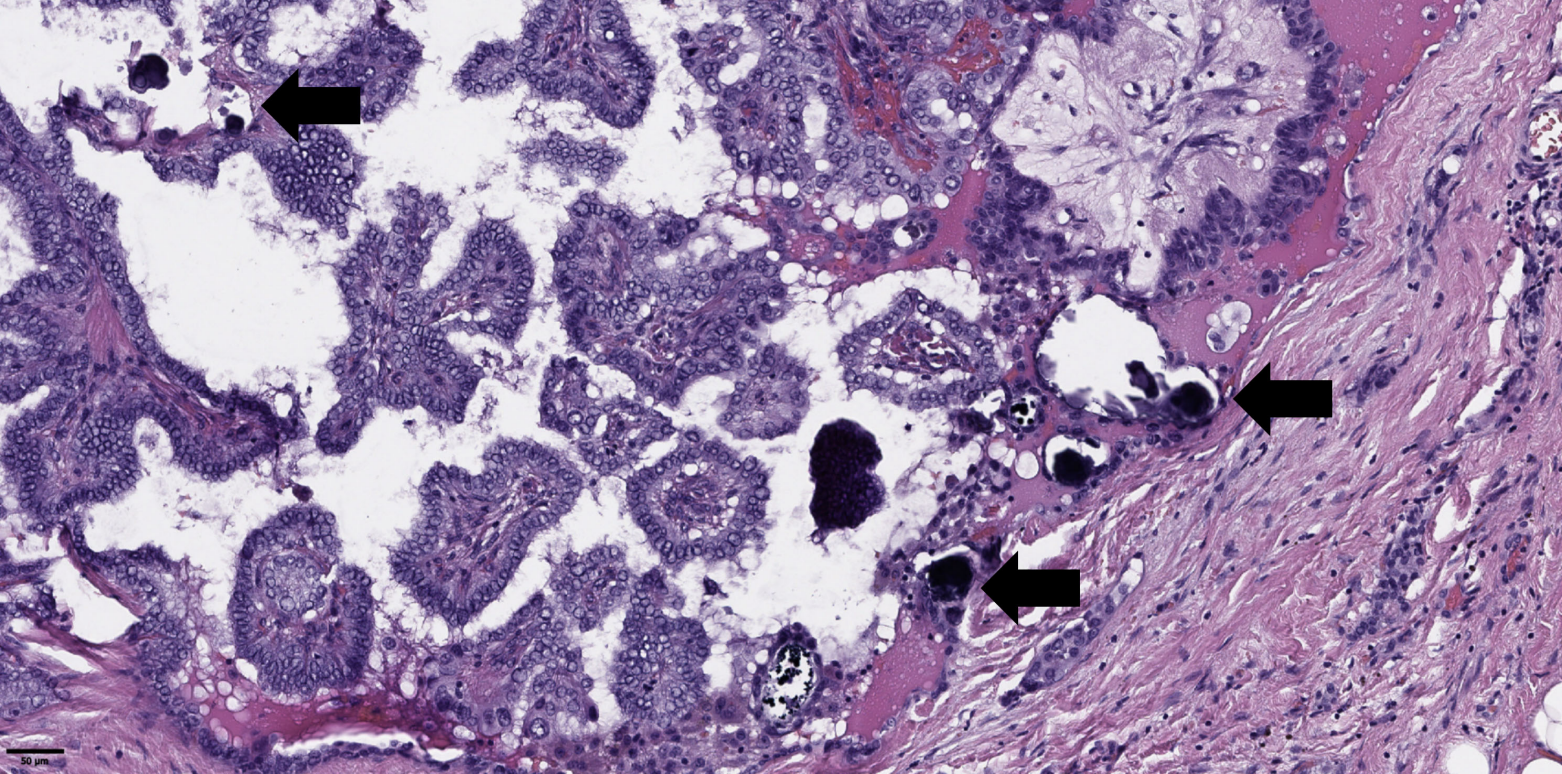
Histological images of psammoma bodies in tumor of papillary thyroid microcarcinoma (black arrows). Hematoxylin–eosin, х100.

**Figure 5:**
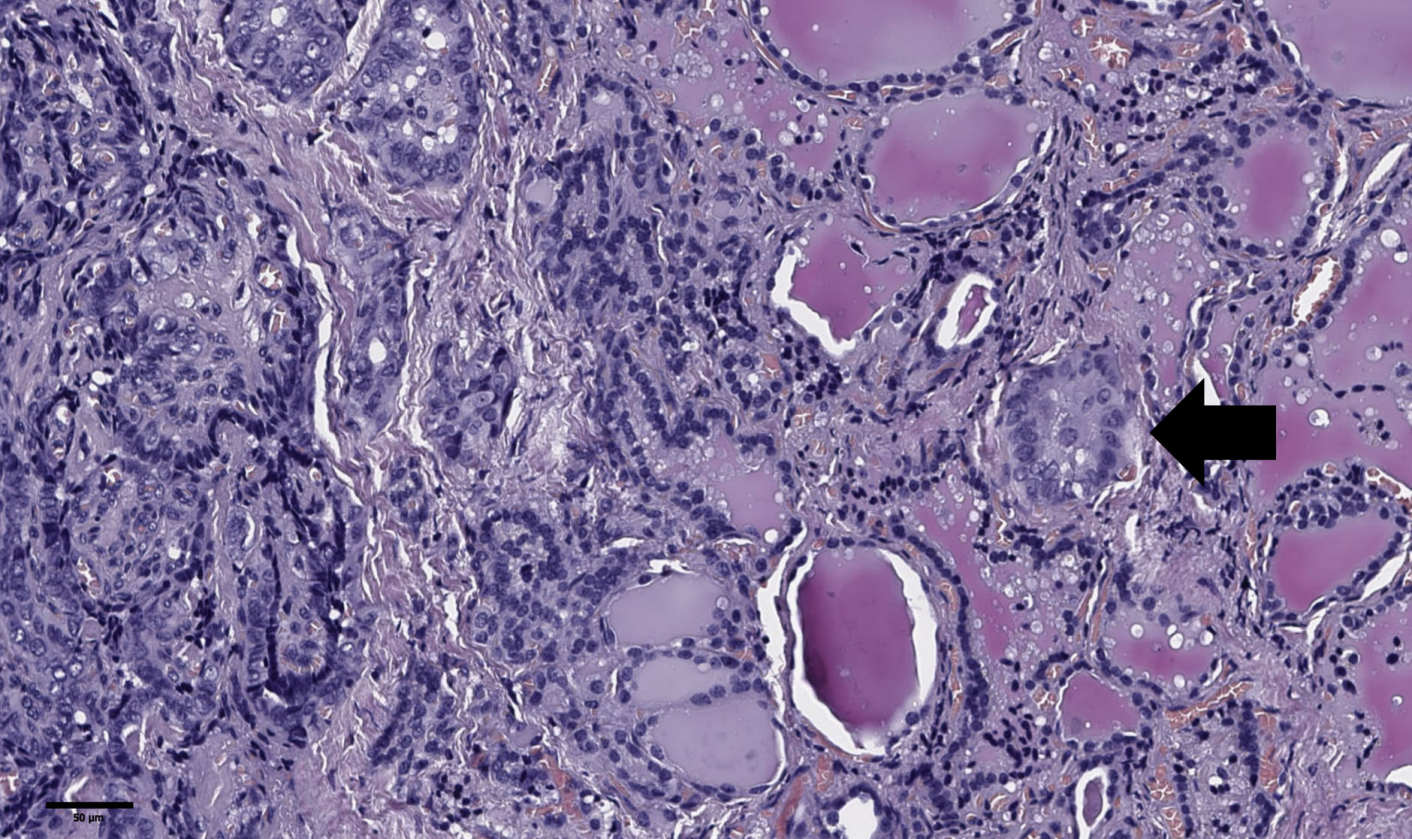
Histological images of tumor “bud” of papillary thyroid microcarcinoma (black arrow). A tumor “bud” was defined as a tumor complex no larger than 0.1 cm in diameter, located no more than 0.1 cm far from the tumor node. Hematoxylin–eosin, х100.

**Figure 6:**
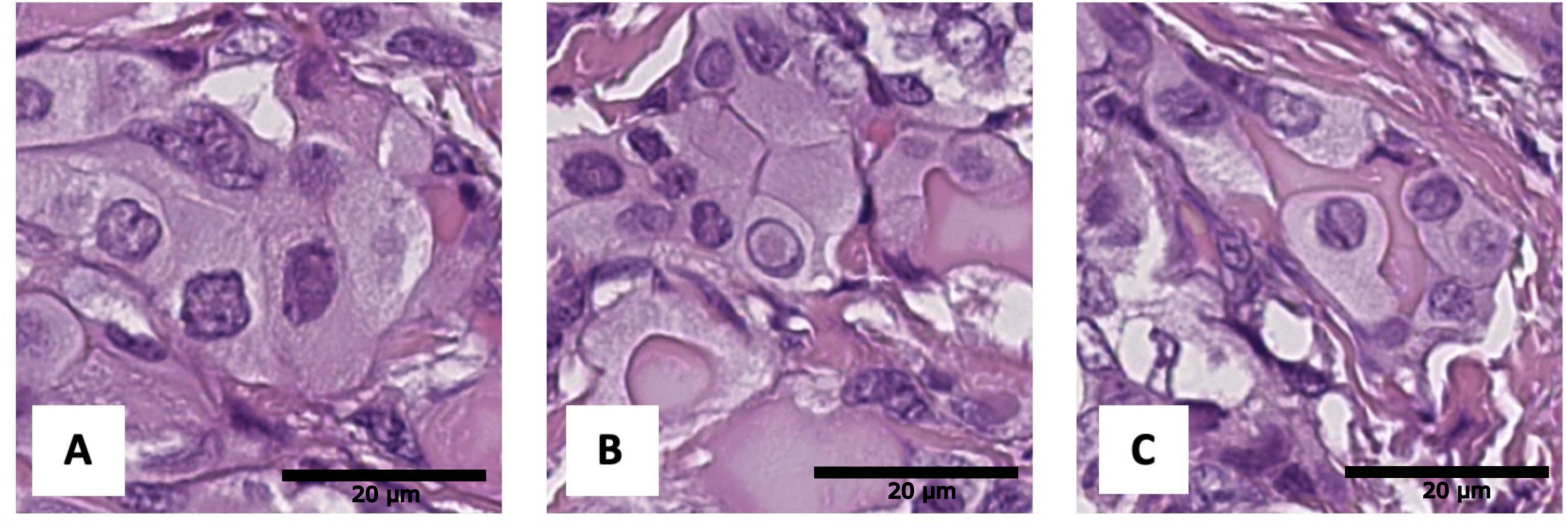
Histological images of tumor nuclear features: **(A)** angular contours, **(B)** intranuclear pseudoinclusions, **(C)** nuclear grooves. Hematoxylin–eosin, х600.

**Figure 7:**
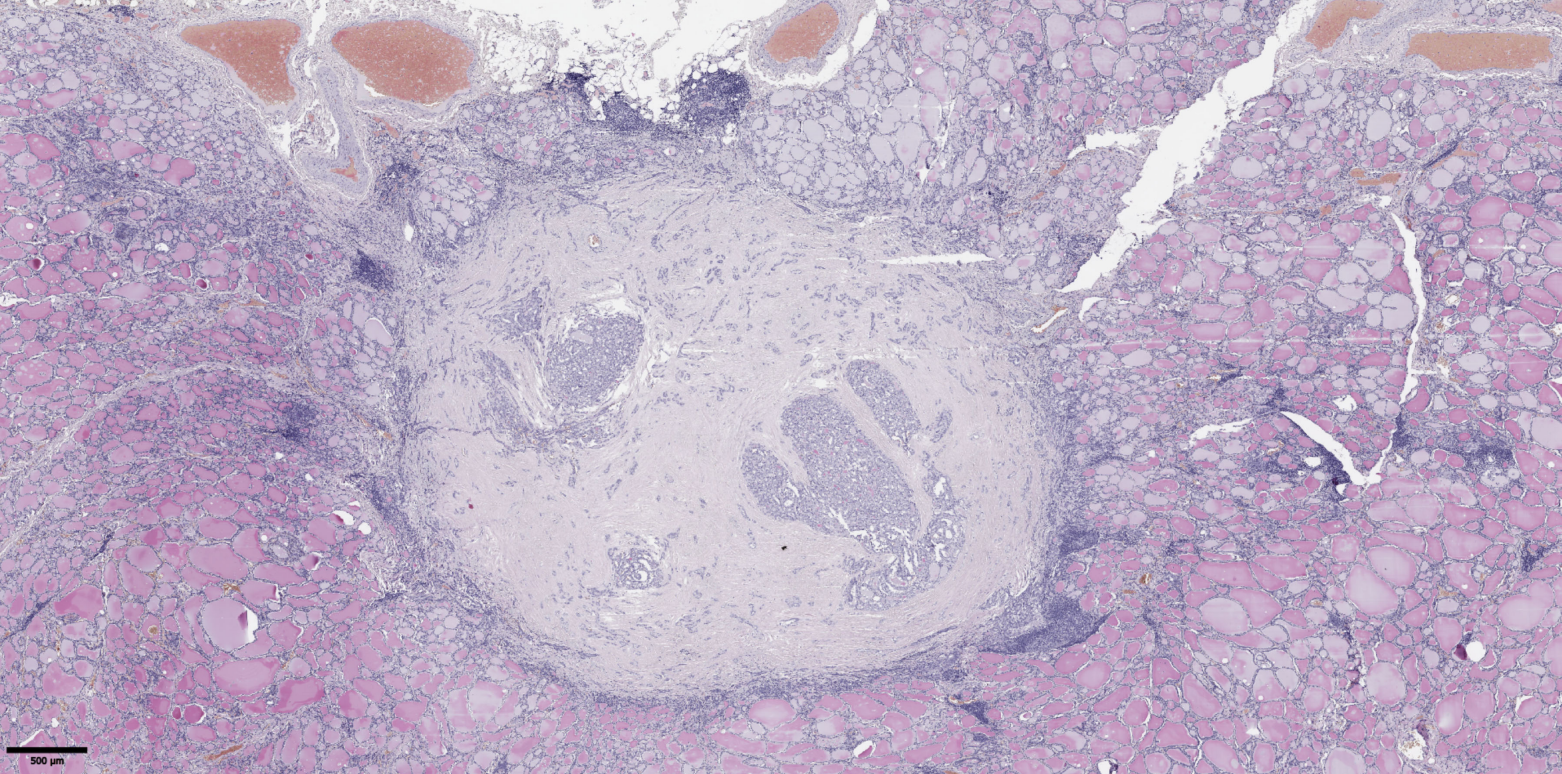
Histological images of papillary thyroid microcarcinoma with fibrosis in 50% or more of the tumor node. Hematoxylin–eosin, х20.

The groups were compared based on a number of tumor morphological features. The following were evaluated: the largest tumor node (**Figure 1**) and metastatic focus size (**Figure 2**), the histological subtype of papillary carcinoma according to the WHO classification (26), the presence of tall cell (**Figure 3**), Warthin-like, and squamous cell components in the tumor, foci of calcification and ossification in the tumor, a lymphocytic rim around the tumor node in the thyroid lobe tissue, apical vacuoles in tumor complexes, psammoma bodies in the tumor tissue (**Figure 4**) and in the thyroid tissue outside the tumor, the presence of a tumor capsule or its fragments, tumor “buds” (**Figure 5**), invasion of adipose tissue, perineural invasion, concomitant nodular thyroid diseases (tumor and non-tumor), and thyroiditis. Additionally, the following nuclear features of tumor cells were noted: angular contours, nuclear grooves, and intranuclear pseudoinclusions (**Figure 6**). Nucleoli in tumor cell nuclei were assessed using criteria partly adopted from the modified WHO/ISUP classification by S.A. Fuhrman for grading renal cell carcinomas (27), namely: the presence of nucleoli basophilic, visible but not prominent at ×100, or nucleoli conspicuous at ×100, basophilic, or nucleoli conspicuous at ×100, eosinophilic at ×400. Furthermore, the number of mitotic figures per 2 mm² of tumor tissue in “hot spots” and the presence of atypical mitotic figures were assessed. The degree of tumor fibrosis was visually graded as 0 (no fibrosis), 1 (fibrosis in less than 50% of the tumor node), or 2 (fibrosis in 50% or more of the tumor node) (**Figure 7**). A tumor “bud” was defined as a tumor complex no larger than 0.1 cm in diameter, located no more than 0.1 cm far from the tumor node, with one or more non-neoplastic thyroid follicles between them, which could not be reliably differentiated during routine histological examination between a tumor embolus in a vessel lumen (blood or lymphatic), an additional independent microfocus of tumor growth, or a tangential section of a tumor invasion area into adjacent thyroid tissue.

Scanning of histological slides was performed using a Leica Aperio AT2 histoscanner (Leica Biosystems) at ×200 magnification. Measurements of tumor “bud” size and their distance from the main tumor node were performed on scanned histological slides using the built-in measurement tool in Aperio eSlide Manager software (version 12.4.3.5008, Leica Biosystems). Histological slides and their digital scans were independently evaluated by three specialists from the Department of Fundamental Pathomorphology of the Endocrinology Research Center (Moscow, Russia). No significant discrepancies in the interpretation of the observed changes were noted; in rare cases involving specific items, decisions were made collectively. No patients were excluded from the study due to a lack of consensus among the experts. The groups were also compared based on the presence of the *BRAF V600E* mutation in tumor tissue, sex, age, and body mass index (BMI) of the patients. BMI was calculated using the standard formula as the ratio of the patient’s weight (in kilograms) to the square of their height (in meters). The *BRAF V600E* mutation was determined by Real-Time PCR using the “Test-BRAF-tissue-24” system (TestGen). The study was performed in 97 out of 100 cases—material from 1 patient in the group without lymph node involvement and 2 patients in the group with lymph node metastases was excluded from the analysis due to severe calcification and “harsh” decalcification of the material. The main characteristics of the overall patient group are presented in **Table 1**.

**Table 1:** Characteristics of the patient group (n=100).

Feature		Total (n=100) Me [Q_1_; Q_3_]/n (%)	Men (n=19) Me [Q_1_; Q_3_]/n (%)	Women (n=81) Me [Q_1_; Q_3_]/n (%)
Age, full years		46 [34; 61]	50 [38; 61]	45 [34; 60]
Largest size of tumor node, cm		0,7 [0.5; 0.9]	0,7 [0.5; 0.9]	0,7 [0.5; 0.9]
Presence of metastases in regional lymph nodes		50 (50%)	11 (58%)	39 (48%)
Largest metastasis, cm		0.3 [0.1; 0.4]	0.4 [0.2; 0.9]	0.3 [0.1; 0.4]
BMI, kg/m²		26 [22; 30]	28 [26; 31]	26 [22; 30]
Type of surgery	Hemithyroidectomy	73 (73%)	11 (58%)	62 (77%)
	Thyroidectomy	23 (23%)	7 (37%)	16 (20%)
	Isthmusectomy	4 (4%)	1 (5%)	3 (4%)
Histological subtype of tumor	Classic	65 (65%)	9 (47%)	56 (69%)
	Infiltrative follicular	13 (13%)	3 (16%)	10 (12%)
	Tall cell	10 (10%)	3 (16%)	7 (9%)
	Encapsulated classic	7 (7%)	2 (11%)	5 (6%)
	Invasive encapsulated follicular	3 (3%)	2 (11%)	1 (1%)
	Warthin-like	2 (2%)	0 (0%)	2 (2%)
Presence of Warthin-like component		3 (3%)	0 (0%)	3 (4%)
Presence of tall cell component		41 (41%)	6 (32%)	35 (43%)
Presence of squamous cell component		5 (5%)	1 (5%)	4 (5%)
Nucleoli basophilic, visible but not prominent at ×100		84 (84%)	15 (79%)	69 (85%)
Nucleoli conspicuous at ×100, basophilic		11 (11%)	2 (11%)	9 (11%)
Nucleoli conspicuous at ×100, eosinophilic at ×400		18 (18%)	3 (16%)	15 (19%)
Angular nuclei		54 (54%)	9 (47%)	45 (56%)
Nuclear grooves		73 (73%)	13 (68%)	60 (74%)
Intranuclear inclusions		33 (33%)	6 (32%)	27 (33%)
Lymphocytic rim around tumor		10 (10%)	0 (0%)	10 (12%)
Apical vacuoles in tumor complexes		62 (62%)	11 (58%)	51 (63%)
Presence of tumor “buds”		48 (48%)	7 (37%)	41 (51%)
Psammoma bodies in tumor		41 (41%)	8 (42%)	33 (41%)
Psammoma bodies outside tumor		21 (21%)	4 (21%)	17 (21%)
Tumor calcification		25 (25%)	5 (26%)	20 (25%)
Tumor ossification		5 (5%)	1 (5%)	4 (5%)
Degree of tumor fibrosis	0 (No)	11 (11%)	3 (16%)	8 (10%)
	1 (<50%)	74 (74%)	13 (68%)	61 (75%)
	2 (≥50%)	15 (15%)	3 (16%)	12 (15%)
Number of mitotic figures per 2 mm² in “hot spots”	0	50 (50%)	6 (32%)	44 (54%)
	1	28 (28%)	7 (37%)	21 (26%)
	2	14 (14%)	2 (11%)	12 (15%)
	3	7 (7%)	4 (21%)	3 (4%)
	4	1 (1%)	0 (0%)	1 (1%)
Atypical mitotic figures		7 (7%)	3 (16%)	4 (5%)
Concomitant oncocytic adenoma		4 (4%)	0 (0%)	4 (5%)
Concomitant follicular adenoma		2 (2%)	0 (0%)	2 (2%)
Concomitant FT-UMP		2 (2%)	0 (0%)	2 (2%)
Concomitant WDT-UMP		1 (1%)	0 (0%)	1 (1%)
Concomitant lymphocytic thyroiditis		43 (43%)	1 (5%)	42 (52%)
Concomitant multinodular hyperplasia		30 (30%)	7 (37%)	23 (28%)
Perineural invasion		3 (3%)	1 (5%)	2 (2%)
Adipose tissue invasion		17 (17%)	4 (21%)	13 (16%)
Presence of tumor capsule elements		54 (54%)	11 (58%)	43 (53%)
*BRAF V600E* mutation		65 (67%)	11 (61%)	54 (68%)

### 2.3 Statistical analysis

Statistical analysis was performed using the Python 3.11 programming language. Descriptive statistics for quantitative features are presented as medians, first and third quartiles in the format Me [Q1; Q3], and categorical features as absolute and relative frequencies in the format n (%). Comparative analysis of two independent groups for quantitative features was performed using the Mann-Whitney U test, and for categorical features using the two-sided Fisher’s exact test (FET2). The critical level of statistical significance was set at 0.05. For multiple comparisons, the Benjamini-Hochberg correction (p0) was applied. Cutoff points for individual parameters were determined using ROC analysis. For cutoff points, odds ratios (OR) with 95% confidence intervals (CI) were calculated. To identify a combination of factors statistically significantly associated with the presence of metastases, logistic regression analysis was performed.

## 3 Results

A comparative analysis of patients with and without metastases in regional lymph nodes is presented in **Table 2**.

**Table 2:** Comparative analysis of patients with and without lymph node metastases.

Feature			Metastases present (n=50)	Metastases absent (n=50)	p	p_0_
			Me [Q_1_; Q_3_]/n (%)	Me [Q_1_; Q_3_]/n (%)		
Sex		Men	11 (22%)	8 (16%)	0.611^a^	0.028
		Women	39 (78%)	42 (84%)		
Age, full years			36 [32; 47]	59 [44; 64]	<0.001^b^	**0.003**
Largest size of tumor node, cm			0.8 [0.5; 1.0]	0.6 [0.4; 0.8]	0.007^b^	**0.008**
BMI, kg/m²			24 [20; 29]	27 [24; 31]	0.007^b^	**0.009**
Histological subtype of tumor	Classic		40 (80%)	25 (50%)	0.003^a^	**0.005**
	Infiltrative follicular		2 (4%)	11 (22%)	0.015^a^	0.012
	Tall cell		6 (12%)	4 (8%)	0.525^a^	0.024
	Encapsulated classic		1 (2%)	6 (12%)	0.112^a^	0.015
	Invasive encapsulated follicular		0 (0%)	3 (6%)	0.242^a^	0.018
	Warthin-like		1 (2%)	1 (2%)	1.000^a^	0.040
Presence of Warthin-like component			2 (4%)	1 (2%)	1.000^a^	0.047
Presence of tall cell component			25 (50%)	16 (32%)	0.103^a^	0.014
Presence of squamous cell component			2 (4%)	3 (6%)	1.000^a^	0.045
Nucleoli basophilic, visible but not prominent at ×100			43 (86%)	41 (82%)	0.786^a^	0.035
Nucleoli conspicuous at ×100, basophilic			4 (8%)	7 (14%)	0.525^a^	0.023
Nucleoli conspicuous at ×100, eosinophilic at ×400			10 (20%)	8 (16%)	0.795^a^	0.036
Angular nuclei			29 (58%)	25 (50%)	0.547^a^	0.026
Nuclear grooves			35 (70%)	38 (76%)	0.653^a^	0.032
Intranuclear inclusions			14 (28%)	19 (38%)	0.395^a^	0.021
Lymphocytic rim around tumor			4 (8%)	6 (12%)	0.741^a^	0.033
Apical vacuoles in tumor complexes			32 (64%)	30 (60%)	0.837^a^	0.038
Presence of tumor “buds”			35 (70%)	13 (26%)	**<0.001** ^a^	**0.001**
Psammoma bodies in tumor			30 (60%)	11 (22%)	**<0.001** ^a^	**0.004**
Psammoma bodies outside tumor			15 (30%)	6 (12%)	0.048^a^	0.013
Tumor calcification			11 (22%)	14 (28%)	0.645^a^	0.031
Tumor ossification			2 (4%)	3 (6%)	1.000^a^	0.046
Degree of tumor fibrosis		0 (No)	1 (2%)	10 (20%)	0.011^a^	0.010
		1 (<50%)	42 (84%)	32 (64%)		
		2 (≥50%)	7 (14%)	8 (16%)		
Number of mitotic figures per 2 mm² in “hot spots”		0	27 (54%)	23 (46%)	0.812^a^	0.037
		1	12 (24%)	16 (32%)		
		2	7 (14%)	7 (14%)		
		3	3 (6%)	4 (8%)		
		4	1 (2%)	0 (0%)		
Atypical mitotic figures			3 (6%)	4 (8%)	1.000^a^	0.041
Concomitant oncocytic adenoma			1 (2%)	3 (6%)	0.617^a^	0.029
Concomitant follicular adenoma			1 (2%)	1 (2%)	1.000^a^	0.042
Concomitant FT-UMP			1 (2%)	1 (2%)	1.000^a^	0.049
Concomitant WDT-UMP			0 (0%)	1 (2%)	1.000^a^	0.044
Concomitant lymphocytic thyroiditis			24 (48%)	19 (38%)	0.419^a^	0.022
Concomitant multinodular hyperplasia			8 (16%)	22 (44%)	**0.004** ^a^	**0.006**
Perineural invasion			2 (4%)	1 (2%)	1.000^a^	0.050
Adipose tissue invasion			10 (20%)	7 (14%)	0.595^a^	0.027
Presence of tumor capsule elements			23 (46%)	31 (62%)	0.160^a^	0.017
*BRAF V600E* mutation			34 из 47 (72%)	31 (62%)	0.291^a^	0.019

a FET2.

b U-test. Bold values means statistically reliable.

The comparative analysis revealed that patients with metastases were younger (p<0.001; p_0_ = 0.003), had a lower BMI (p=0.007; p_0_ = 0.009), and a larger tumor node size (p=0.007; p_0_ = 0.008). The classic subtype of carcinoma (p=0.003; p_0_ = 0.005), psammoma bodies in tumor tissue (p<0.001; p_0_ = 0.004), and tumor “buds” (p<0.001; p_0_ = 0.001) were more frequently identified in these patients. No metastases were found in patients with tumor node sizes <0.3 cm. Concomitant multinodular hyperplasia was less common in patients with metastases compared to those without (p=0.004; p_0_ = 0.006). Patients with metastases also showed a statistical trend toward more pronounced tumor fibrosis (p=0.011; p_0_ = 0.010) and more frequent detection of psammoma bodies in thyroid tissue outside the tumor (p=0.048; p_0_ = 0.013). In patients without metastases, a statistical trend toward more frequent detection of the infiltrative follicular tumor subtype was observed (p=0.015; p_0_ = 0.012).

Next, odds ratios (OR) were calculated for features that showed statistically significant differences. The classification matrix for patients with and without metastases based on these features is presented in **Table 3**.

**Table 3:** Classification matrix for patients with and without metastases based on features showing statistically significant differences.

Feature	Metastases present (n=50)	Metastases absent (n=50)
Age < 52 лет	42	16
Age ≥ 52 лет	8	34
Largest size of tumor node ≥ 0.7 см	35	23
Largest size of tumor node < 0.7 см	15	27
BMI < 22.9 kg/m²	21	6
BMI ≥ 22.9 kg/m²	29	44
Classic histological subtype	40	25
Non-classic histological subtype	10	25
Tumor “buds” present	35	13
Tumor “buds” absent	15	37
Psammoma bodies in tumor present	30	11
Psammoma bodies in tumor absent	20	39
Concomitant multinodular hyperplasia present	8	22
Concomitant multinodular hyperplasia absent	42	28

**Figure 8:**
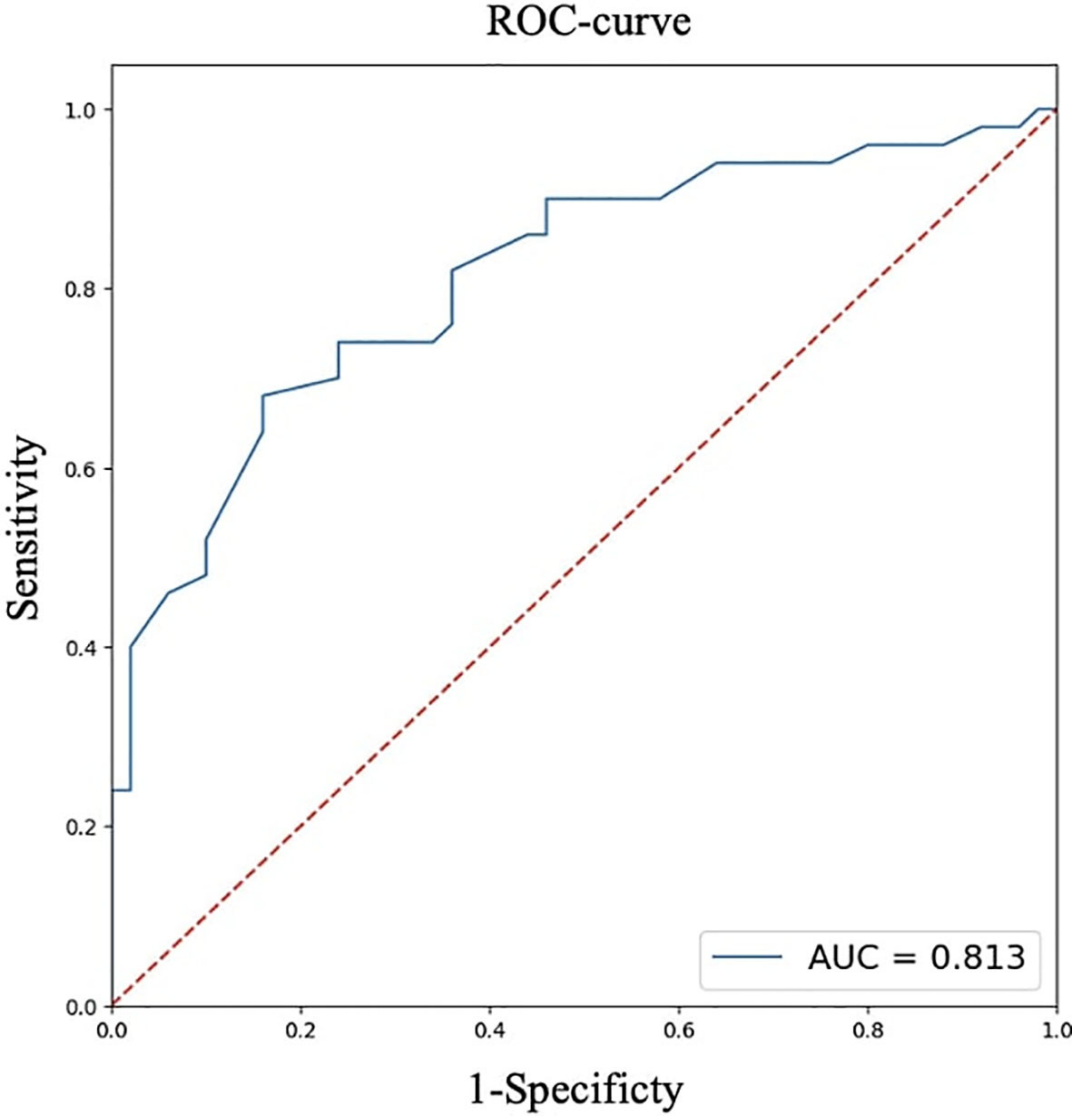
ROC analysis of age.

**Figure 9:**
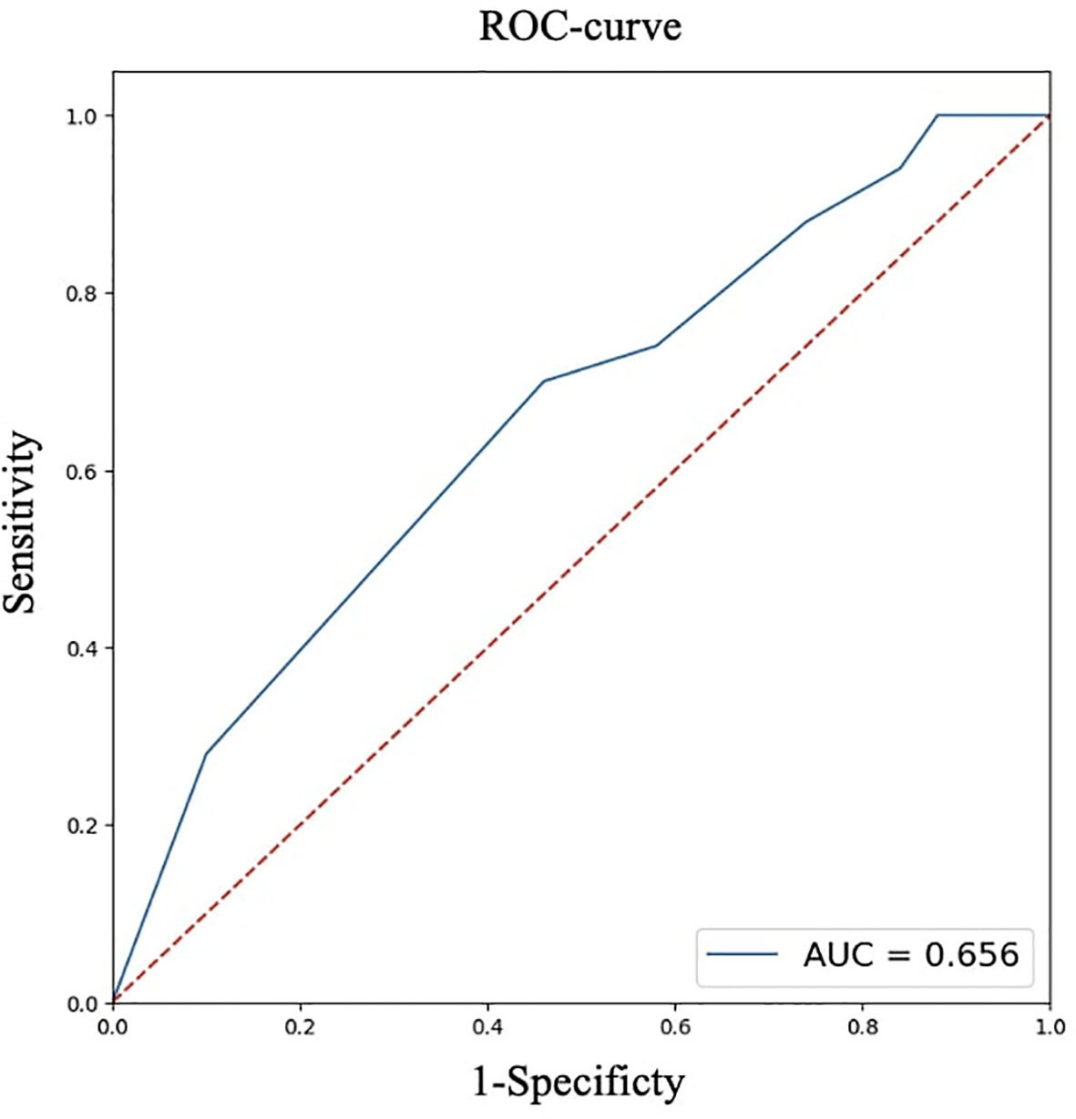
ROC analysis of largest size of tumor node.

**Figure 10:**
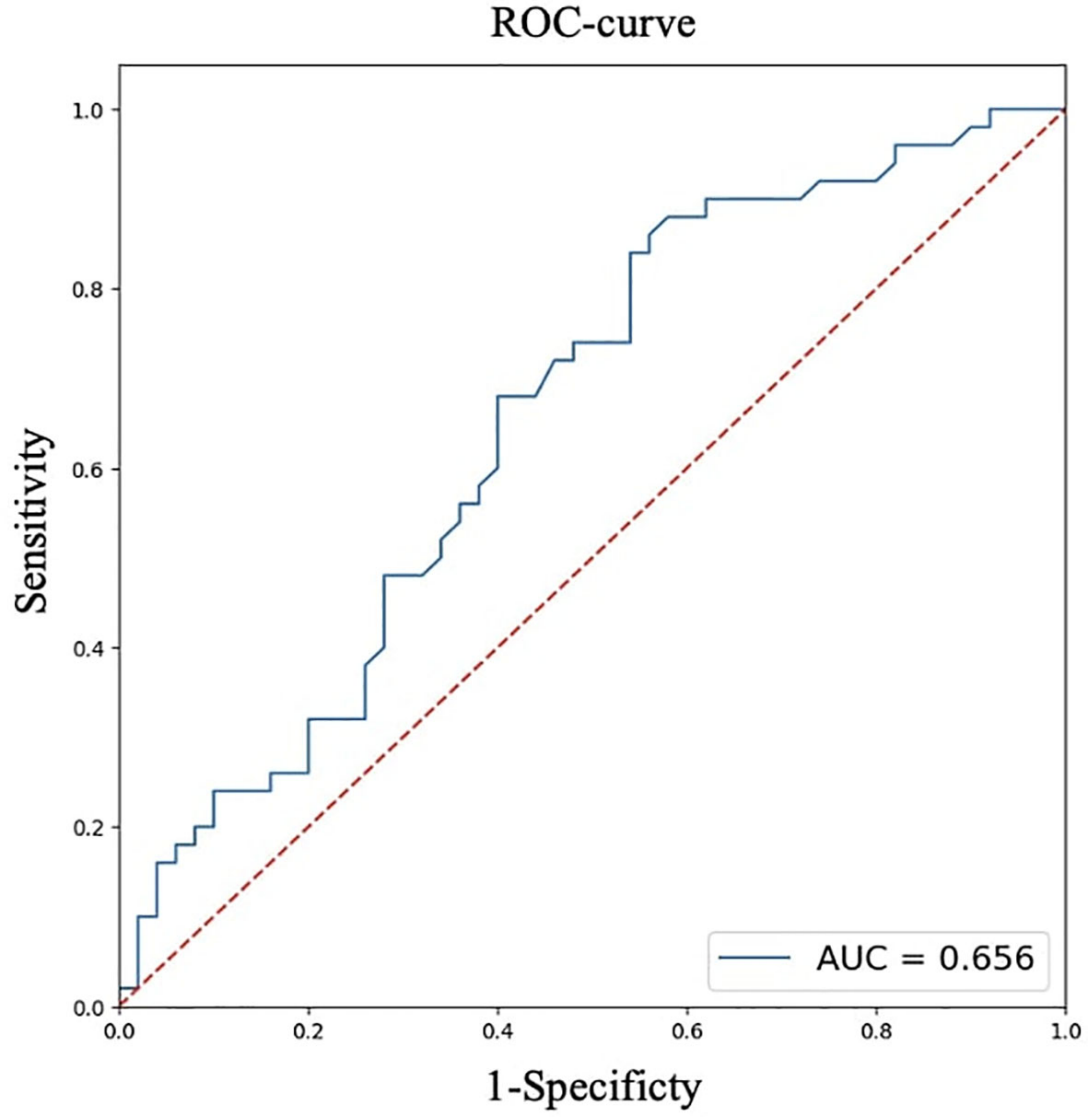
ROC analysis of BMI.

**Figure 11:**
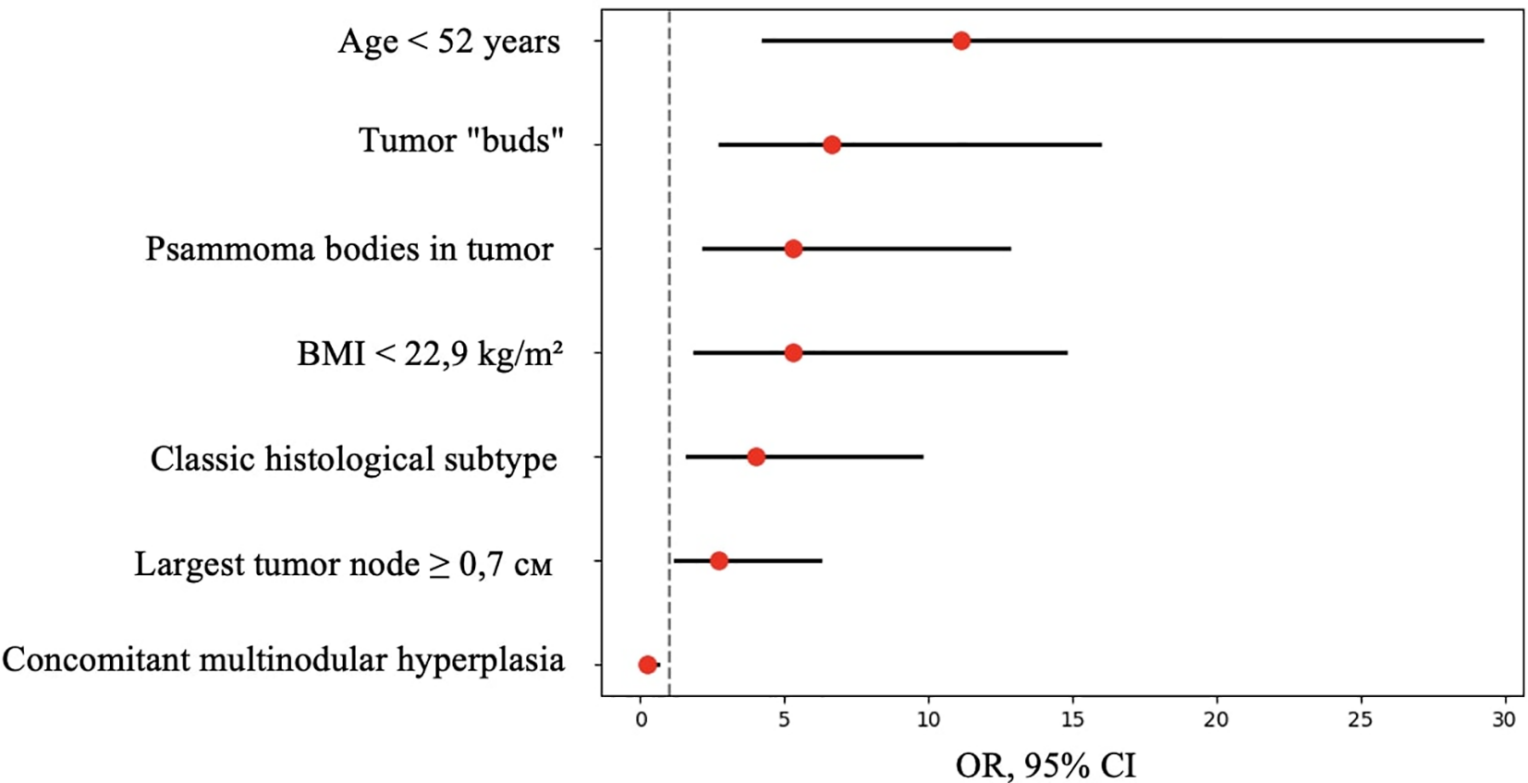
OR, 95% CI for features showing statistically significant differences between patients with and without metastases.

**Figure 12:**
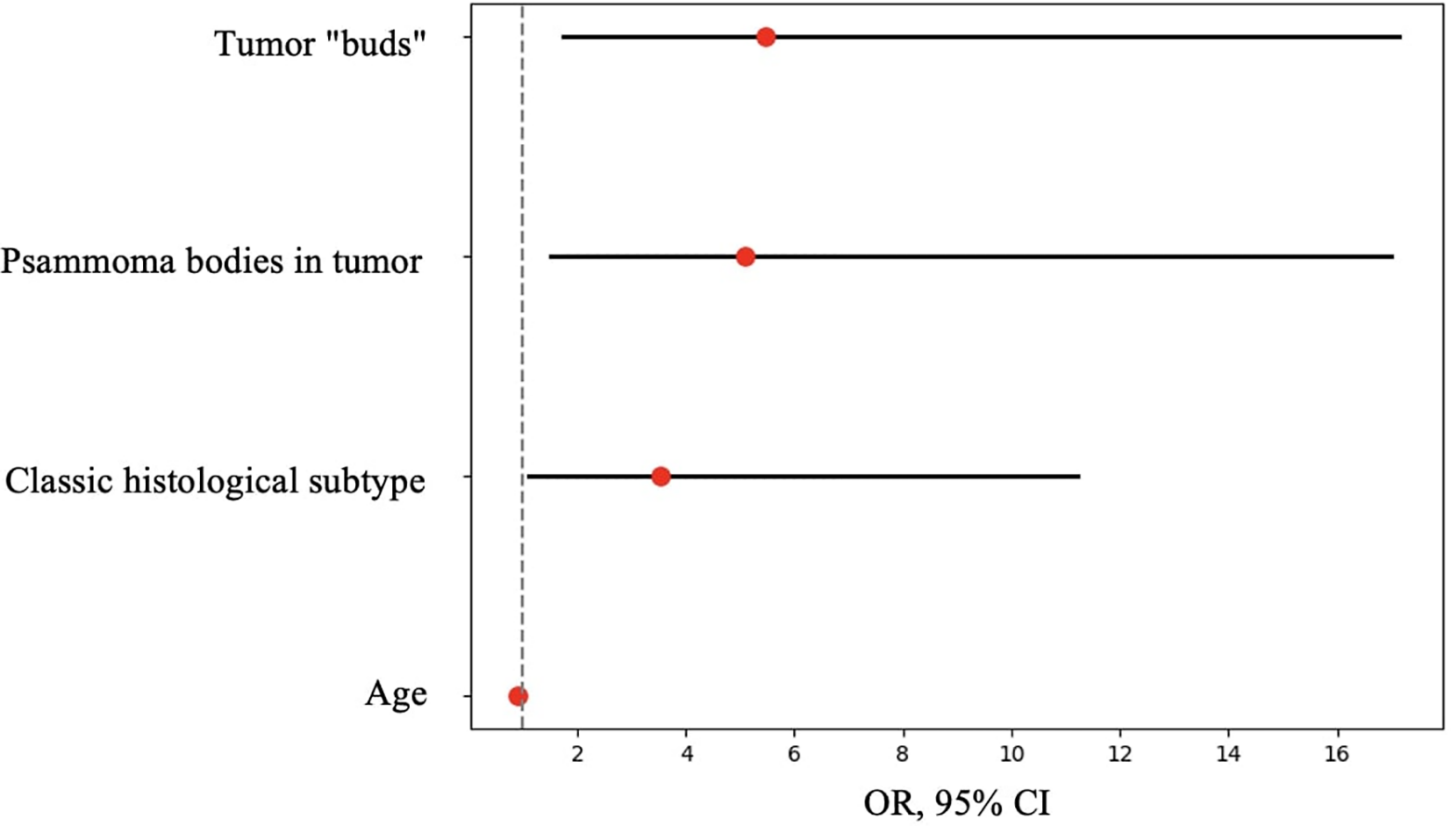
OR, 95% CI for parameters included in the logistic regression model.

For quantitative features, ROC analysis was preliminarily performed (**Table 4**, **Figures 8**–**12**).

**Table 4:** ROC analysis for quantitative features.

Parameters	Age, full years	Largest size of tumor node, cm	BMI, kg/m²
AUC	0.813	0.656	0.656
95% CI (AUC)	0.728-0.898	0.548-0.762	0.549-0.763
Cutoff point (Youden index)	52	0.7	22.9
OR	11.156	2.739	5.310
95% CI (OR)	4.265-29.184	1.204-6.230	1.913-14.745

Thus, patients younger than 52 years had a 4.3–29.2 times higher chance of having metastases compared to older patients. For tumor node sizes ≥0.7 cm, the chance of metastases was 1.2–6.2 times higher than for smaller tumors. Patients with a BMI <22.9 kg/m² had a 1.9–14.7 times higher chance of metastases compared to those with a higher BMI.

Based on the obtained data (**Table 5**), patients with the classic tumor subtype had a 1.6–9.7 times higher chance of metastases compared to other subtypes. The presence of tumor “buds” increased the chance of detecting metastases by 2.7–15.9 times, and patients with psammoma bodies in tumor tissue had a 2.2–12.8 times higher chance of metastases compared to those without. Conversely, patients with concomitant multinodular hyperplasia had a 1.6–10.5 times lower chance of metastases compared to those without it.

**Table 5:** Odds ratios (OR) for qualitative features.

Parameters	OR	95% CI
Classic tumor subtype	4.000	1.647-9.715
Presence of tumor “buds”	6.641	2.769-15.927
Presence of psammoma bodies	5.318	2.214-12.774
Concomitant multinodular hyperplasia	0.242	0.095-0.621

Next, logistic regression analysis was performed to identify a combination of factors associated with the presence of metastases. All parameters that showed statistical significance in the comparative analysis were analyzed as predictors:

- Age, years
- BMI, kg/m²
- Largest size of tumor node, cm
- Classic tumor subtype (yes/no)
- Tumor “buds” (yes/no)
- Psammoma bodies in tumor tissue (yes/no)
- Concomitant multinodular hyperplasia (yes/no)

The response variable was the presence of metastases (yes/no). The results identified a combination of parameters statistically significantly associated with the presence of metastases (**Table 6**).

**Table 6:** Characteristics of parameters included in the logistic regression model.

Parameters	Beta	OR, 95% CI	р, Wald test
Age	-0.103	0.902 (0.859-0.946)	<0.001
Classic tumor subtype	1.259	3.521 (1.106-11.205)	0.033
Tumor “buds”	1.700	5.476 (1.749-17.141)	0.003
Psammoma bodies in tumor	1.625	5.079 (1.517-17.004)	0.008
Constant	2.635		0.020

Thus, the presence of a classic tumor subtype, tumor “buds,” and psammoma bodies in tumor tissue increased the chance of metastases by 3.5, 5.5, and 5 times, respectively. Each additional year of age reduced the chance of metastases by 10% of the current value.

A comparative analysis of patients with and without the *BRAF V600E* mutation is presented in **Table 7**.

**Table 7:** Comparative analysis of patients with and without the *BRAF V600E* mutation.

Feature		Mut *BRAF V600E* + (n=65)	Mut *BRAF V600E* – (n=32)	p	p_0_
		Me [Q_1_; Q_3_]/n (%)	Me [Q_1_; Q_3_]/n (%)		
Sex	Men	11 (17%)	7 (22%)	0.586^a^	0.027
	Women	54 (83%)	25 (78%)		
Age, full years		46 [35; 60]	46 [34; 62]	0.602^b^	0.029
Largest size of tumor node, cm		0.70 [0.50; 0.90]	0,65 [0.50; 0.90]	0.804^b^	0.037
Presence of metastases in regional lymph nodes		34 (52%)	13 (41%)	0.291^a^	0,017
BMI, kg/m²		26 [22; 29]	27 [23; 32]	0.397^b^	0.022
Histological subtype of tumor	Classic	42 (65%)	20 (62%)	1.000^a^	0.046
	Infiltrative follicular	6 (9%)	7 (22%)	0.114^a^	0.009
	Tall cell	10 (15%)	0 (0%)	0.014^a^	0.003
	Encapsulated classic	6 (9%)	1 (3%)	0.420^a^	0.023
	Invasive encapsulated follicular	0 (0%)	3 (9%)	0.034^a^	0.004
	Warthin-like	1 (2%)	1 (3%)	1.000^a^	0.038
Presence of Warthin-like component		2 (3%)	1 (3%)	1.000^a^	0.045
Presence of tall cell component		35 (54%)	4 (12%)	**<0**.**001** ^a^	**0**.**001**
Presence of squamous cell component		2 (3%)	3 (9%)	0.328^a^	0.018
Nucleoli basophilic, visible but not prominent at ×100		58 (89%)	24 (75%)	0.080^a^	0.006
Nucleoli conspicuous at ×100, basophilic		7 (11%)	3 (9%)	1.000^a^	0.050
Nucleoli conspicuous at ×100, eosinophilic at ×400		12 (18%)	6 (19%)	1.000^a^	0.042
Angular nuclei		38 (58%)	13 (41%)	0.131^a^	0.010
Nuclear grooves		50 (77%)	23 (72%)	0.622^a^	0.031
Intranuclear inclusions		22 (34%)	10 (31%)	1.000^a^	0.047
Lymphocytic rim around tumor		4 (6%)	6 (19%)	0.077^a^	0.005
Apical vacuoles in tumor complexes		43 (66%)	18 (56%)	0.377^a^	0.021
Presence of tumor “buds”		35 (54%)	12 (38%)	0.139^a^	0.012
Psammoma bodies in tumor		28 (43%)	12 (38%)	0.665^a^	0.033
Psammoma bodies outside tumor		15 (23%)	5 (16%)	0.439^a^	0.026
Tumor calcification		15 (23%)	8 (25%)	1.000^a^	0.044
Tumor ossification		1 (2%)	3 (9%)	0.103^a^	0.008
Degree of tumor fibrosis	0 (No)	6 (9%)	5 (16%)	0.154^a^	0.013
	1 (≥50%)	52 (80%)	20 (62%)		
	2 (≥50%)	7 (11%)	7 (22%)		
Number of mitotic figures per 2 mm² in “hot spots”	0	32 (49%)	15 (47%)	0.705^a^	0.036
	1	19 (29%)	9 (28%)		
	2	10 (15%)	4 (12%)		
	3	4 (6%)	3 (9%)		
	4	0 (0%)	1 (3%)		
Atypical mitotic figures		6 (9%)	1 (3%)	0.420^a^	0.024
Concomitant oncocytic adenoma		2 (3%)	2 (6%)	0.597^a^	0.028
Concomitant follicular adenoma		2 (3%)	0 (0%)	1.000^a^	0.041
Concomitant FT-UMP		1 (2%)	1 (3%)	1.000^a^	0.049
Concomitant WDT-UMP		0 (0%)	1 (3%)	0.330^a^	0.019
Concomitant lymphocytic thyroiditis		27 (42%)	15 (47%)	0.667^a^	0.035
Concomitant multinodular hyperplasia		16 (25%)	13 (41%)	0.156^a^	0.014
Perineural invasion		2 (3%)	1 (3%)	1.000^a^	0.040
Adipose tissue invasion		13 (20%)	3 (9%)	0.250^a^	0.015
Presence of tumor capsule elements		37 (57%)	16 (50%)	0.665^a^	0.032

a FET2.

b U-test. Bold values means statistically reliable.

Analysis of the *BRAF V600E* mutation revealed its presence in 65 out of 97 patients in the overall group (11 men, 54 women). Patients with the *BRAF V600E* mutation had a significantly higher frequency of tall cell tumor components (p<0.001; p_0_ = 0.001). There was also a statistical trend toward a higher frequency of the tall cell tumor subtype (p=0.014; p_0_ = 0.003) and a lower frequency of the invasive encapsulated follicular subtype (p=0.034; p_0_ = 0.004) compared to patients without the mutation.

## 4 Discussion and conclusions

Thus, according to the univariate analysis of the overall patient group, an increased risk of synchronous regional lymph node metastases in unifocal papillary thyroid microcarcinoma was observed in patients with the classic tumor subtype, the presence of tumor “buds,” psammoma bodies in tumor tissue, and the absence of concomitant multinodular hyperplasia. Additionally, risk factors included a tumor node size ≥0.7 cm, BMI <22.9 kg/m², and age <52 years. However, in the multivariate analysis, only the presence of tumor “buds,” psammoma bodies in tumor tissue, and patient age showed significant associations with the risk of synchronous regional lymph node metastases. Therefore, in our definition tumor “buds” may indeed be a sign of more aggressive invasive tumor growth, intraorgan dissemination, or lymphatic vessel invasion. The possibility of *de novo* development of additional tumor foci cannot be entirely ruled out. Despite the identified morphological and clinical predictors of metastasis, the significance of these findings in the context of disease prognosis remains debatable, as overall and recurrence-free survival rates for papillary thyroid microcarcinoma remain extremely high (28, 29). In this study, we did not aim to determine the nature of tumor “buds” in each specific case. Further research using 3D visualization, immunohistochemical markers (e.g., CD31, D2-40), molecular profiling, and sequencing could clarify their nature—whether they are emboli, microfoci, tangential sections, or invasion areas. However, this process may be labor-intensive, costly, and not always successful due to the small size of such complexes (≤0.1 cm). Nevertheless, the term “tumor bud” seems appropriate as a risk factor detectable during routine histological examination. It should be noted that the term “tumor budding” is currently not used in the context of thyroid carcinomas. In histological evaluation of colorectal adenocarcinomas, it refers to single cells or small clusters composed of fewer than five cells located at the invasive front of the tumor (30). Therefore, the definition of tumor “budding” adopted in our study is novel and differs from the definition used in studies of colorectal carcinomas.

Regarding the higher frequency of psammoma bodies in tumor tissue and the statistical trend toward more frequent detection of psammoma bodies outside the tumor in patients with metastases, similar findings have been reported in large studies by Bai et al. and Pyo et al. (31, 32). Unfortunately, Bai et al. did not specify whether psammoma bodies were detected in tumor tissue or in thyroid tissue outside the tumor. Additionally, the study group in that work consisted of patients with tumor nodes ≥1 cm (mostly ≥2 cm). Pyo et al. included patients with papillary microcarcinomas as well as larger tumors, and approximately 30% of the overall group had multifocal tumor growth. In that study, the presence of psammoma bodies in both tumor tissue and thyroid tissue outside the tumor showed statistical significance for regional metastasis detection. Unfortunately, in our study, the detection of psammoma bodies outside the tumor showed only a statistical trend toward more frequent synchronous metastasis detection. Moreover, there are differences in statistical methods. However, the association between psammoma bodies in tumor tissue and regional lymph node metastases was confirmed in both univariate and multivariate analyses. Furthermore, our findings align well with the results of J.V. Johannessen and M. Sobrinho-Simões, who suggested that psammoma bodies may represent calcified papillary tumor structures or tumor emboli in lymphatic vessels (33). Additionally, Liu et al. (34) noted that intra- and extratumoral microcalcifications detected during ultrasound examinations of patients with papillary thyroid cancer are risk factors for cervical lymph node metastases. The association between patient age and the risk of synchronous regional lymph node metastases identified in our study also correlates well with the results of Liu et al.: we found an increased risk of metastases in patients younger than 52 years, while Liu et al. reported an increased risk in patients younger than 55 years.

Tumor size is a criterion in most staging systems for differentiated thyroid cancer (35), including AJCC TNM (36). In our study, a tumor node size ≥0.7 cm emerged as a risk factor, but only in univariate analysis. It is noteworthy that no metastatic lymph node involvement was observed in patients with tumor sizes <0.3 cm. The identified association between patient BMI and the risk of regional lymph node metastasis is of particular interest. Recently, excess body weight has been considered a risk factor for papillary thyroid cancer development (1, 37). According to the large MASTER study published in 2023, obesity was associated with the frequency of extrathyroidal extension and the development of intermediate-risk papillary thyroid carcinomas in women (38). There is also evidence of a correlation between increased BMI and the development of papillary thyroid carcinomas >4 cm in size (39). Meanwhile, J.M. Kim reported a possible association between overweight/obesity and multifocal papillary thyroid cancer growth and recurrence but did not note a link with tumor size, extrathyroidal extension, or regional lymph node metastasis development (40). Our study found a significant association between lower BMI and an increased risk of regional lymph node metastasis, but only in univariate analysis.

In this study, we attempted to use nucleolar features of tumor cells—similar to those employed in the modified WHO/ISUP classification by S.A. Fuhrman—as a potential predictor of metastasis. It is known, for instance, that the original S.A. Fuhrman criteria have also been applied in the L.M. Weiss classification developed for adrenocortical tumors (41). The criteria we developed were conceptually similar to the original classification: we also assessed the basophilia and eosinophilia of nucleoli within the nuclei of tumor cells at ×100 and ×400 magnification. However, significant differences existed in the specific details of the criteria. This is due to difficulties we encountered in directly applying the original criteria from the modified S.A. Fuhrman classification, which is widely used for grading renal cell carcinoma. These difficulties arose from the predominance of basophilic nucleoli and the absence of well-defined eosinophilic nucleoli at ×100 magnification in cells of papillary thyroid microcarcinoma.

Unfortunately, this study did not identify any correlation between the structural features of nucleoli in tumor cells and the presence of regional lymph node metastases, nor with the BRAF V600E mutation. Besides, the presence of the *BRAF V600E* mutation in tumor tissue showed no association with the frequency of regional metastasis detection. However, patients with this mutation had a higher frequency of tall cell tumor components and a statistical trend toward a higher frequency of the tall cell tumor subtype and a lower frequency of the invasive encapsulated follicular subtype—consistent with current data on molecular alterations in various papillary carcinoma subtypes. It is known that the high-cell subtype of papillary thyroid microcarcinoma has a >92% frequency of the *BRAF V600E* mutation, while the invasive encapsulated follicular subtype is primarily associated with mutations in genes such as RAS and PAX8/PPARγ (42).

Interestingly, in 2023, our colleagues from a hospital in Shandong Province (China) published a study focused on risk factors for metastasis in papillary thyroid carcinoma, the results of which partially contradict our own findings (43). Among other factors, they identified age ≥45 years, BMI ≥25, and the presence of the BRAF V600E mutation as risk factors. Several notable differences between our studies may account for the observed discrepancies in results. From a research standpoint, the strengths of the Chinese study include a significantly larger sample size (400 patients) and the routine performance of central lymph node dissection in accordance with Chinese clinical guidelines for the diagnosis and treatment of differentiated thyroid carcinoma. This allowed for pathologic assessment of lymph node metastases in all patients. Despite this, the proportion of patients with lymph node metastases was lower (35%). Their cohort also included patients with multifocal tumor growth patterns and more than 20% of patients had tumors measuring ≥1 cm. Papillary thyroid carcinoma was not subtyped in that study.

In our study, the control group included patients without clinical evidence of lymph node metastases in the neck, and, in accordance with Russian clinical guidelines for the treatment of differentiated thyroid carcinoma, central lymph node dissection was not routinely performed. However, individual lymph nodes closely adjacent to the thyroid tissue may have been removed during surgery. One must also take into account the substantial ethnic differences between the patient populations studied.

Among the results we have obtained, the higher frequency of the classic tumor subtype among patients with metastases is noteworthy, as, according to current understanding, patients with this subtype are not typically classified as high-risk. It is possible that histological subtyping is more subjective for papillary microcarcinomas than for larger tumors, and the challenges increase with smaller tumor sizes. Therefore, it may be worthwhile to conduct a study assessing the degree of interobserver agreement in histological subtyping of papillary thyroid microcarcinomas. It cannot be ruled out that some features distinguishing non-classic subtypes are minimally represented in small tumors, raising questions about the necessity of precise subtyping for such cases. Additionally, the prognostic significance of gene expression profiles in tumor tissue may be more relevant for papillary microcarcinomas than for larger tumors, but this requires separate investigation.

## Data Availability

The original contributions presented in the study are included in the article/supplementary material. Further inquiries can be directed to the corresponding author.
